# Uterus-Preserving Management of Intractable Haemorrhage Following Suction and Evacuation: A Case Series

**DOI:** 10.7759/cureus.81606

**Published:** 2025-04-02

**Authors:** Ritu Singh, Poonam Lal, Swaroop R Nanda, Monika Gupta, Avinash K Singh

**Affiliations:** 1 Obstetrics and Gynaecology, Kurji Holy Family Hospital, Patna, IND; 2 Radiodiagnosis, Indira Gandhi Institute of Medical Sciences, Patna, Patna, IND

**Keywords:** bipolar uterine artery coagulation, intractable hemorrhage, laparoscopic management in pph, laparoscopy, molar pregnancy, pregnancy, secondary pph, suction evacuation, uterine artery ligation, uterus conservation

## Abstract

Suction and evacuation (S&E) is a very common procedure in obstetric patients with minimal blood loss. Although rare, in cases of secondary postpartum haemorrhage (PPH) due to retained products of conception (RPOC) following delivery (vaginal or caesarean section) and in hydatidiform mole, there is a chance of intractable haemorrhage, following S&E, which may require a hysterectomy. Hysterectomies not only lead to premature menopause but also increase overall morbidity and risk of mortality to the patient. The objective of this study is to highlight the occurrence of intractable haemorrhage following S&E in cases of secondary PPH and molar pregnancies and to evaluate uterine-preserving surgical techniques as effective alternatives to hysterectomy.

We present three cases of intractable bleeding following S&E that were managed with conservative uterine preservation. The first case was a 26-year-old primipara presented with secondary PPH one month post-caesarean, requiring blood transfusion. Ultrasound revealed RPOC, and she underwent suction evacuation, but massive bleeding occurred. Suspecting uterine perforation, an exploratory laparotomy was performed, revealing a normal uterus. Bilateral uterine artery ligation successfully controlled the haemorrhage, preserving the uterus. The patient had an uneventful recovery and was discharged on day 7.

The second case was a 30-year-old female (Para 3, Live Births 2) with a history of two lower segment caesarean sections (LSCS), who presented with heavy postpartum bleeding 21 days after a preterm vaginal birth after caesarean section (VBAC). Ultrasound revealed RPOC, and she underwent S&E, but heavy bleeding persisted despite uterotonics. Laparoscopic bilateral uterine artery coagulation successfully controlled the bleeding, preserving the uterus. The patient recovered well and was discharged on postoperative day 3.

In the third case, a 38-year-old female (Para 5, Live Births 3, Deaths 2) with a diagnosed molar pregnancy underwent S&E, during which she developed a massive haemorrhage. Despite medical management and uterine massage, the bleeding persisted. Laparoscopic uterine artery coagulation successfully controlled the haemorrhage, preserving the uterus. The patient received three units of packed red blood cells (PRBC), recovered well, and was discharged on postoperative day 3, with weekly monitoring until beta-human chorionic gonadotropin (β-hCG) levels normalized.

For the conservation of the uterus, uterine artery ligation via laparotomy or laparoscopic coagulation of the uterine artery are both effective and safe procedures. In primigravida patients or those desiring future pregnancies, bilateral uterine artery ligation can be performed to save the uterus, either by laparotomy or laparoscopy, depending on the surgeon’s expertise. Laparoscopy offers the added advantages of smaller incisions and a shorter hospital stay.

## Introduction

Suction and evacuation (S&E) is a very common procedure in obstetric patients after abortion with minimal blood loss. India's abortion rate stands at 47.0 abortions (42.2-52.1) per 1,000 women aged 15-49 years annually, with 2.2 million (14%) of these being surgical abortions [1], mainly suction evacuation procedures. Complications of the surgical procedure may include uterine infection, injury, perforation, sepsis, and shock [1].

Although rare, in cases of secondary postpartum haemorrhage (PPH) due to retained products of conception (RPOC) following delivery (vaginal or caesarean section) [2] and evacuation of hydatidiform mole, there is a chance of intractable haemorrhage [3]. This intractable bleeding can lead to shock, anemia, the need for blood transfusions, and, in rare cases, may require a hysterectomy [4]. A recent study by Chainarong et al. conducted over 14 years on 123 cases of secondary haemorrhage found that two (1.6%) required a hysterectomy for placental tissue with intractable bleeding [2]. There are also case reports of intractable bleeding following suction evacuation of hydatidiform mole, requiring hysterectomy [3].

Hysterectomies not only lead to premature menopause but also increase overall morbidity and risk of mortality to the patient. According to a recent study published in the *American Journal of Obstetrics and Gynecology*, women who undergo hysterectomy, even with ovarian preservation, have an increased risk of all-cause mortality compared to those who have not undergone surgery (hazard ratio: 1.29; 95% confidence interval: 1.19-1.40) [5].

The objective of the study is to highlight the occurrence of intractable haemorrhage following S&E in cases of secondary PPH and molar pregnancies and to evaluate uterine-preserving surgical techniques as effective alternatives to hysterectomy. So, we present three cases of intractable bleeding following S&E that were managed with conservative methods for uterine preservation.

## Case presentation

The first case was a primipara who developed secondary PPH after a caesarean section. She was managed with open bilateral uterine artery ligation. The second case was a multipara with two previous lower segment caesarean sections (LSCS), who had a preterm vaginal birth after caesarean section (VBAC) this time. The third case involved a molar pregnancy. Both the second and third cases were managed by laparoscopic bilateral uterine artery coagulation.

Case 1

A 26-year-old female primipara presented to our hospital with a complaint of continuous vaginal bleeding for the past two days, following a caesarean section performed one month ago at another hospital. She received two units of blood for secondary PPH before presenting to our hospital. She was hemodynamically stable, with a pallor. Her baseline blood investigations are shown in Table 1.

**Table 1:** Laboratory investigation of Case 1

Lab Parameter	Lab Values	Reference Range
Haemoglobin	6.6 g/dL	12-16 g/dL
Total leukocyte count	13.11 x10^3^ cells/µL	5.2-12.4x 10^3 ^cells/µL
Platelet	172 x10^3 ^cells/µL	140-400x10^3 ^cells/µL
Serum bilirubin direct/total	0.19/0.38 mg/dL	0.5-1.5/0-0.6 mg/dL
Alanine aminotransferase (ALT)	11 U/L	0-40 U/L
Prothrombin time with international normalized ratio (INR)	17 sec/1.3	11-14 sec/0.9-1.3
Random blood sugar	173 mg/dL	75-139 mg/dL
Urea	13 mg/dL	10-50 mg/dL
Creatinine	0.4 mg/dL	0.6-1.4 mg/dL
Serum sodium	135.95 mEq/L	135-145 mEq/L
Serum potassium	3.84 mEq/L	3.5-5.0 mEq/L

Her haemoglobin level was 6.6 g/dL. Ultrasound revealed a bulky uterus measuring 10 × 5 cm, with a hypoechoic collection of 4.2 × 3.7 cm, showing minimal internal vascularity, suggestive of RPOC. There is no evidence of an arteriovenous malformation. Two units of blood were crossmatched and arranged, and the patient underwent suction evacuation. Although the uterus appeared empty after S&E, she started bleeding massively. Despite the administration of uterotonics and vigorous uterine massage, the bleeding did not stop. Suspecting uterine perforation, we proceeded with an exploratory laparotomy with consent for a hysterectomy. Upon opening the abdomen, the uterus appeared normal, and bilateral uterine artery ligation was performed, which successfully controlled the bleeding. Afterwards, we repeated S&E, removing a minimal amount of tissue, and we could preserve the uterus. Two units of blood were transfused during the procedure, and one unit was given postoperatively. Her postoperative period was uneventful, with postoperative haemoglobin of 10 g/dL. The patient was discharged after seven days.

**Figure 1:**
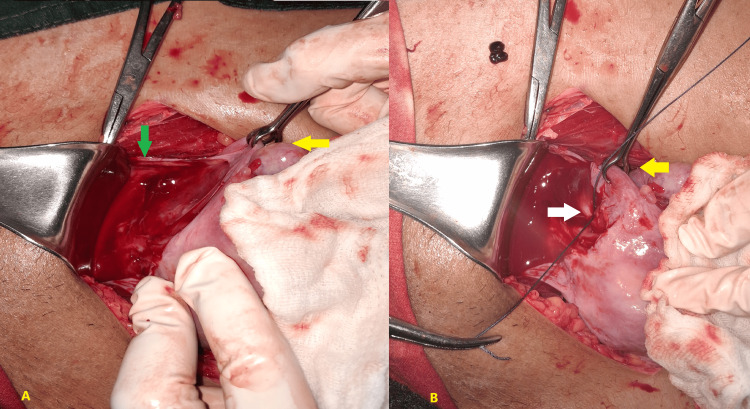
Open uterine artery ligation (A) The yellow arrow indicates the round ligament grasped with Babcock’s forceps. The green arrow shows the broad ligament anterior to the uterus being opened. (B) The suture is tied anteriorly. The white arrow highlights the uterine vessels.

In bilateral uterine artery ligation, the round ligament is grasped with Babcock forceps, and the broad ligament anterior to the uterus is opened, with the bladder pushed downward as shown in Figure 1A. The first needle is passed from anterior to posterior through the uterine musculature at the level of the isthmus, 1 to 2 centimeters medial to the uterine vessels. The needle is then passed again from posterior to anterior, lateral to the uterine vessels, through the broad ligament in the avascular space medial to the round ligament. The suture is tied anteriorly, as shown in Figure 1B. The same procedure is repeated on the other side.

Case 2

A 30-year-old female (Para 3, Live Births 2) with a history of two previous LSCS (13 years and 11 years back) presented to the gynecology outpatient department (OPD) with postpartum heavy bleeding that started 21 days after a VBAC. In third pregnancy, she had preterm vaginal delivery at 29 weeks, with a baby weighing 1.5 kg, who cried at birth but passed away after two days. The patient reported excessive bleeding during delivery, although no blood transfusion was required at that time. However, 21 days postpartum, she began experiencing heavy bleeding again and underwent S&E at another hospital, where she received one unit of packed red blood cells (PRBC). Despite this, the bleeding persisted, and she was referred to our hospital for further management. On examination, her pulse was 86/min, blood pressure was 100/60 mmHg, and she exhibited pallor. Abdominal examination was soft and non-tender, and per vaginal examination revealed a closed cervical os with no active bleeding at that time. Her investigations are shown in Table 2.

**Table 2:** Laboratory investigation of Case 2

Lab Parameter	Lab Values	Reference Range
Haemoglobin	6.2 g/dL	12-16 g/dL
Total leukocyte count	5.8x10^3^ cells/µL	5.2-12.4x 10^3 ^cells/µL
Platelet	204x10^3 ^cells/µL	140-400x10^3 ^cells/µL
Beta-human chorionic gonadotropin (β-hCG)	16.17 mIU/mL	<5 mIU/mL
Serum bilirubin direct/total	0.1/0.2 mg/dL	0.5-1.5/0-0.6mg/dL
Alanine aminotransferase (ALT)	10 U/L	0-40 U/L
Prothrombin time with international normalized ratio (INR)	13/1.0 sec	11-14 sec
Random blood sugar	117 mg/dL	75-139 mg/dL
Urea	13 mg/dL	10-50 mg/dL
Creatinine	0.4 mg/dL	0.6-1.4 mg/dL
Serum sodium	137 mEq/L	135-145 mEq/L
Serum potassium	4.2 mEq/L	3.5-5.0 mEq/L

**Figure 2:**
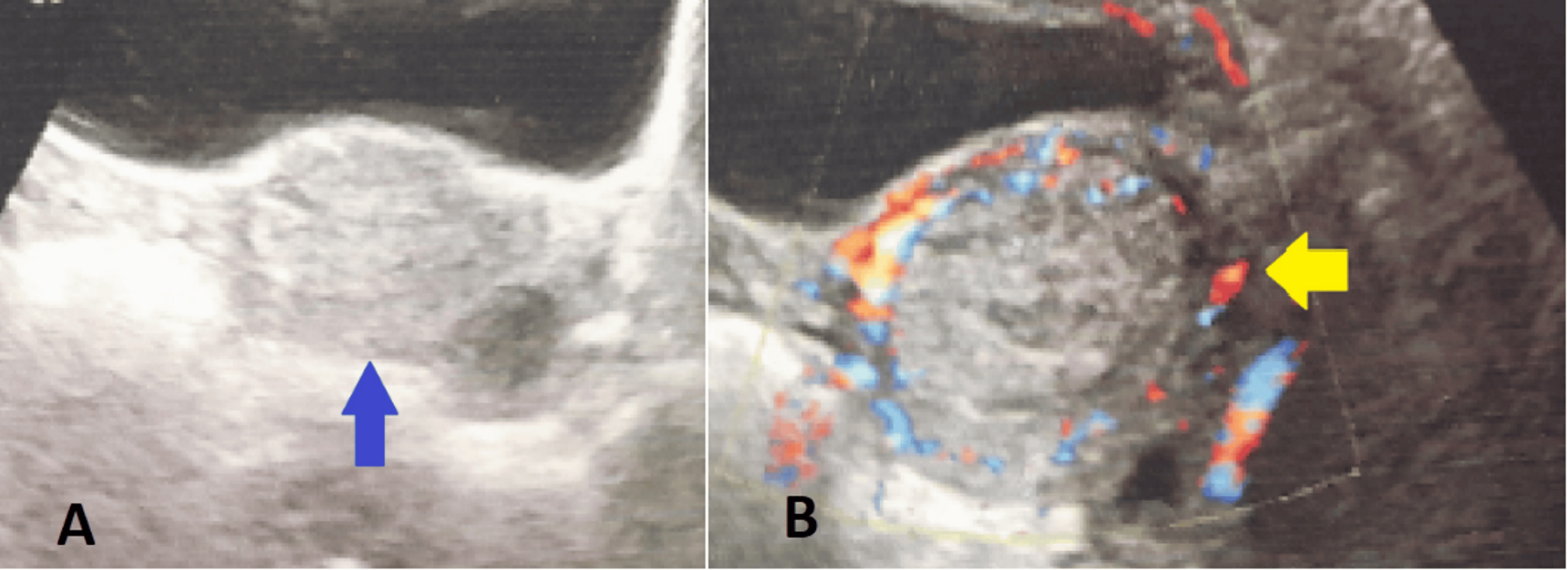
Ultrasound imaging (A) The blue arrow indicates a complex hyperechoic collection in the endometrial cavity measuring 31 × 24 mm. (B) The yellow arrow highlights the peripheral hypoechoic rim with minimal internal vascularity on Doppler imaging.

Her haemoglobin was 6.2 g/dL. An ultrasound showed RPOC measuring 3.2 × 3.7 cm and revealed a complex hyperechoic collection in the endometrial cavity measuring 31 × 24 mm. The peripheral hypoechoic rim and minimal internal vascularity on Doppler imaging were suggestive of RPOC, without evidence of an arteriovenous malformation, as shown in Figure 2.

The patient was posted for S&E with consent for possible laparoscopy, laparotomy, or hysterectomy if necessary. During the procedure, the patient had uncontrolled haemorrhage in spite of uterotonics and uterine massage. A total of four units of PRBC were transfused. Laparoscopic bilateral uterine coagulation was performed, which controlled the bleeding significantly. The procedure for coagulation is described in detail following the third case. Then we did a check curettage, and the uterus was found to be empty. Therefore, the uterus was conserved. Her postoperative period was uneventful overall, with no bleeding in the postoperative period. Her postoperative haemoglobin level was 9.1 g/dL, and other investigations were normal. The patient was discharged three days after the operation.

Case 3

A 38-year-old female (Para 5, Live Births 3, Deaths 2) presented to the OPD with a history of amenorrhea for the past three months. She had no current complaints but brought an ultrasound report showing a molar pregnancy, for which she was seeking S&E. One month prior, she had used a Medical Termination of Pregnancy (MTP) kit, followed by heavy bleeding lasting four days, which was medically managed. Her obstetric history includes two full-term stillbirths, and she had three children of 13, 11, and 6 years old.

On examination, her pulse was 86/min, blood pressure was 100/60 mmHg, and pallor was noted. Abdominal examination revealed a uterus sized at 14-16 weeks, and per vaginal examination confirmed a 16-week-sized anteverted, anteflexed uterus with no active bleeding. Laboratory results are shown in Table 3.

**Table 3:** Laboratory investigation of Case 3

Lab Parameter	Lab Values	Reference Range
Haemoglobin	7.4 g/dL	12-16 g/dL
Total leukocyte count	10.7 x 10^3^ cells/µL	5.2-12.4x 10^3 ^cells/µL
Platelet	229 x10^3 ^cells/µL	140-400x10^3 ^cells/µL
Beta-human chorionic gonadotropin (β-hCG)	32141 mIU/mL	<5 mIU/mL
Serum bilirubin direct/total	0.1/0.6 mg/dL	0.5-1.5/0-0.6 mg/dL
Alanine aminotransferase (ALT)	21 U/L	0-40 U/L
Prothrombin time with international normalized ratio (INR)	16 sec/1.2	11-14 sec/0.9-1.3
Random blood sugar	80 mg/dL	75-139 mg/dL
Urea	40 mg/dL	10-50 mg/dL
Creatinine	0.4 mg/dL	0.6-1.4 mg/dL
Serum sodium	139 mEq/L	135-145 mEq/L
Serum potassium	4.1 mEq/L	3.5-5.0 mEq/L
Serum thyroid-stimulating hormone (TSH)	2.4 mIU/L	0.4-4.5 mIU/L
Free triiodothyronine (T3)	2.4 pg/mL	1.7-3.7 pg/mL
Free thyroxine (T4)	1.4 ng/dL	0.8-1.8 ng/dL

Her haemoglobin was 7.4 g/dL and a beta human chorionic gonadotrophin (β-hCG) level of 32,141 mIU/mL. Her ultrasound showed a bulky uterus of size 11.8 x 5.6 cm with 9.4 x 5.7 cm clusters of small cysts suggestive of molar pregnancy. The patient was taken for S&E after taking consent for excessive bleeding, perforation, and hysterectomy. During the S&E procedure, the patient started having a massive haemorrhage. Despite medical management, bimanual massage, and adequate suctioning, the bleeding did not stop. Laparoscopic uterine artery coagulation was performed after obtaining consent explaining the risks and benefits of the procedure. We had successfully controlled the bleeding and conserved the uterus. A total of three PRBCs was given. The patient tolerated the procedure well. Her postoperative haemoglobin was 10 g/dL. She was discharged three days after the operation. The patient was monitored weekly till her β-hCG level became normal.

**Figure 3:**
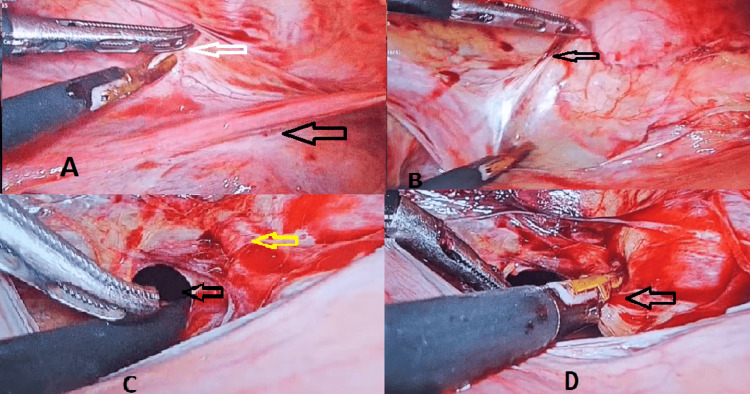
Laparoscopic uterine artery coagulation (A) The black arrow indicates the round ligament, while the white arrow highlights the anterior leaf of the broad ligament. (B) The black arrow shows the anterior leaf of the broad ligament separated from the other side. (C) The black arrow marks a window created in the posterior leaf of the broad ligament, and the yellow arrow highlights the exposed uterine artery and vein complex. (D) The arteriovenous bundle is coagulated using LigaSure (Medtronic, Dublin, Ireland).

The procedure was performed as follows: General anaesthesia was administered to the patient, and a Foley catheter was inserted into the bladder. A pneumoperitoneum was created after inserting a trocar at the umbilicus, and a video laparoscope was introduced into the abdominal cavity. Three 5-mm trocars were then placed in the right and left lower abdomen and the left upper abdomen. The patient was positioned in Trendelenburg so that the intestines were displaced away from the pelvis. The first step was to open the anterior leaf of the broad ligament just below the round ligament, as shown in Figure 3A. The uterus was then gently shifted to the opposite side, and the tissues were carefully separated from the other side, as shown in Figure 3B. At the level of the isthmus, a window was created by cutting the posterior leaf of the broad ligament, which exposed the uterine artery and vein complex, as shown in Figure 3D. The bipolar cautery was used to coagulate the entire arterio-venous bundle, as shown in Figure 3D. The same procedure was then repeated on the other side.

## Discussion

In the literature, the incidence of secondary PPH is reported to range from 0.21% to 2.5% [2,4,6]. Common causes of secondary PPH include retained placenta, endometritis, and delayed placental bed involution [7]. Less common causes are congenital coagulopathies, uterine pseudoaneurysm, vessel subinvolution at the placental implantation site, and uterine rupture [8]. RPOCs are the leading cause of secondary PPH, accounting for 21.1% to 30% of cases [2,7]. Women who deliver via caesarean section have a lower rate of RPOC compared to those who deliver vaginally (10.8% vs. 32.8%) [2]. In our cases, one patient had RPOC following a caesarean section, while the other developed it after a preterm vaginal delivery.

In a study by Chainarong et al., 29.3% of women with secondary PPH required uterine evacuation, and 8.1% needed a hysterectomy [2]. However, an earlier study of 135 patients reported that 63% required surgical evacuation, with one case requiring a hysterectomy [9]. Secondary PPH often necessitates evacuation and may lead to hysterectomy. In our report, we successfully avoided hysterectomy in three cases by performing uterine artery ligation via laparotomy in one case and laparoscopic bipolar uterine artery coagulation in the other two.

In our cases, a blood transfusion was required in each instance. Previous studies also reported blood transfusions in 17-29.3% of cases [2,9]. The median number of blood transfusions in these studies was 2 (1-15) units [2].

Previous studies have shown that the mean age of presentation to the hospital for secondary PPH is typically in the second week after delivery [2,6,9]. However, in our cases, both patients presented after three to four weeks, which may be due to the fact that the studies referenced show a mean age of presentation.

In our initial case, we first suspected perforation as the cause of excessive bleeding. In cases of perforation, laparotomy is also required, and uterine-preserving surgeries can be performed. This is supported by the case report of Khan et al. [10].

In our second case, the patient had a history of primary PPH during her preterm delivery, which could have contributed to the secondary PPH. A study by Hoveyda and MacKenzie also found that a history of primary PPH and manual removal of the placenta were significant risk factors for secondary PPH [9].

A recent systematic review by Fox et al. highlighted that there are no randomized trials on secondary PPH, according to the Cochrane Review [4]. Additionally, the Royal College of Obstetricians and Gynaecologists has not recommended any specific treatment options for secondary PPH [4], underlining the need for further research in this area.

When available, UAE is an alternative treatment in secondary PPH [2], particularly for patients wishing to preserve fertility; however, it can have adverse effects on fertility and reproductive outcomes.

In hydatidiform mole, the most common complications following S&E include excessive bleeding and uterine perforation [11]. The risk of bleeding increases with the size of the uterus [12], and for uteri larger than 16 weeks gestational size, blood transfusions should be readily available [13]. In our case, the uterus was sized at 16 weeks. When available, S&E should be performed with ultrasound guidance to aid in the complete removal of molar tissue and minimize the risk of uterine perforation [13,14]. The procedure is typically done under general anesthesia [15], and an intravenous infusion of oxytocin is often initiated at the start of suction dilation and curettage (D&C) and continued for several hours afterward to improve uterine contractions and reduce blood loss [13]. Although there are theoretical concerns regarding the routine use of oxytocic agents due to the risk of embolizing and disseminating trophoblastic tissue through the venous system, oxytocin is still recommended for managing molar pregnancies [13,15,16]. A medication-only method for uterine evacuation is not recommended due to its high failure rates, risk of haemorrhage, increased chances of post-molar gestational trophoblastic neoplasia (GTN), and greater maternal morbidity [15,17].

A case report on laparoscopic coagulation of the uterine artery for secondary haemorrhage was published earlier [18]. Laparoscopic coagulation has also been used in cases of arteriovenous malformation [19] and symptomatic myomas [20]. In our case series, we demonstrate that uterine artery occlusion, whether performed via laparoscopy or laparotomy, is effective not only in managing secondary PPH but also in controlling intractable haemorrhage following S&E in molar pregnancies.

In a study by Chen et al., the subsequent pregnancy rates following bipolar coagulation of the uterine artery were 41.6% (15/36) for pregnancies and 5.6% (2/36) for term pregnancies, and the authors did not recommend this procedure for women who wish to have additional children [20]. In contrast, previous studies have shown that uterine artery ligation for PPH does not appear to compromise patients' subsequent fertility or obstetric outcomes [21]. In our two cases, where laparoscopic coagulation was performed, both patients were multipara, and we proceeded with coagulation after obtaining consent. For the primipara in our first case, uterine ligation was performed to preserve the uterus.

Our cases have significant implications for enhancing reproductive outcomes, reducing surgical morbidity, and broadening treatment options for managing obstetric haemorrhage.

## Conclusions

Secondary PPH and molar pregnancies, though not very common, can lead to intractable haemorrhage following S&E. For the conservation of the uterus, uterine artery ligation via laparotomy or laparoscopic coagulation of the uterine artery are both effective and safe procedures. In primigravida patients or those desiring future pregnancies, bilateral uterine artery ligation can be performed to save the uterus, either by laparotomy or laparoscopy, depending on the surgeon’s expertise.
